# Supplementary figures and images for: Family caregiver experience of caring COVID-19 patients admitted in COVID-19 hospital of a tertiary care hospital in Nepal
Source: PLoS One. 2024 Jan 5;19(1):e0295395. doi: 10.1371/journal.pone.0295395 (PMC10769026; doi:10.1371/journal.pone.0295395)

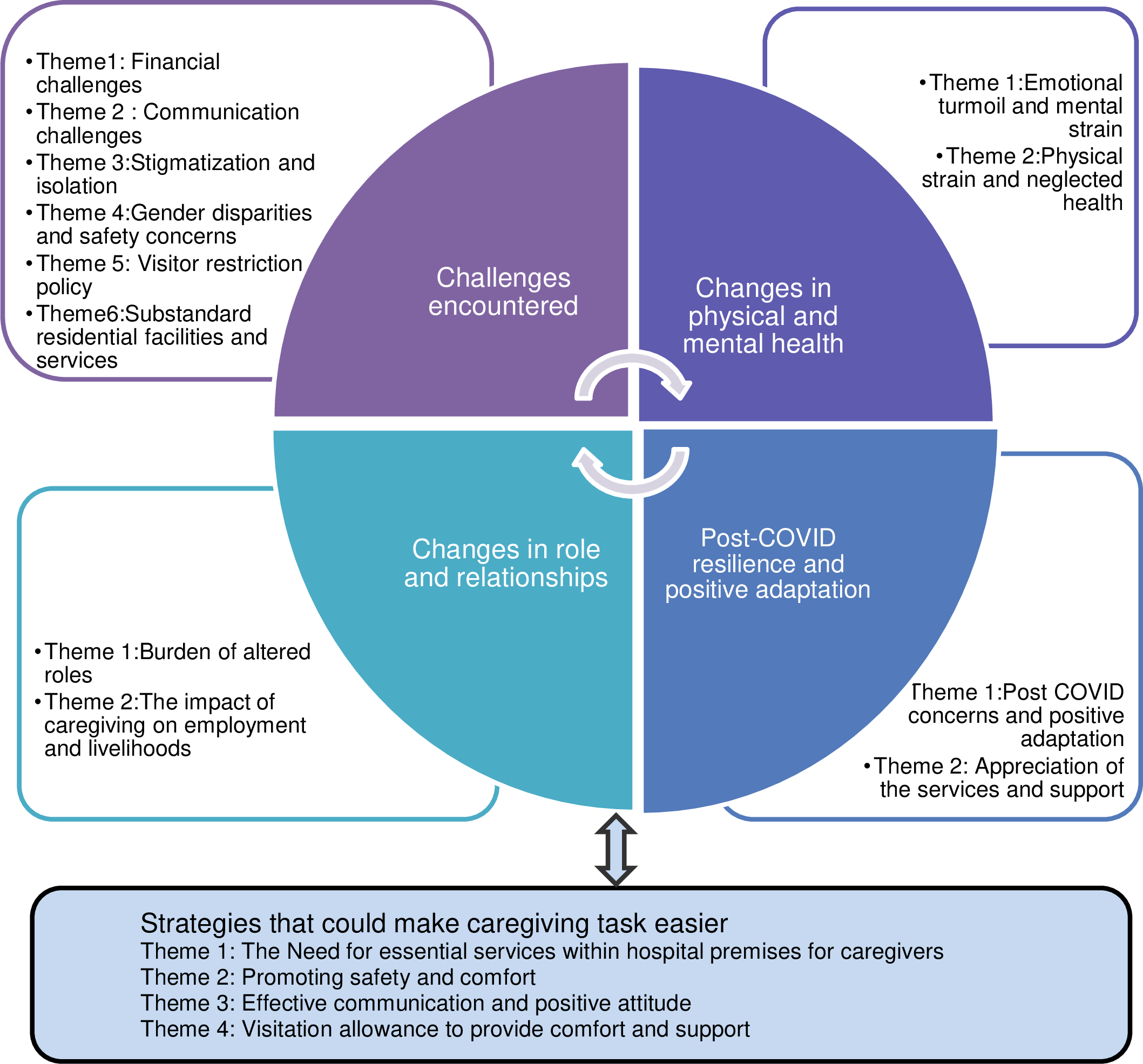

Supplement: S1 Fig — The arrow depicts the way in which each domain is related with other. (TIF) [file pone.0295395.s001.tif]
