# Supplementary material for: Family caregiver experience of caring COVID-19 patients admitted in COVID-19 hospital of a tertiary care hospital in Nepal
Source: PLoS One. 2024 Jan 5;19(1):e0295395. doi: 10.1371/journal.pone.0295395 (PMC10769026; doi:10.1371/journal.pone.0295395)

#
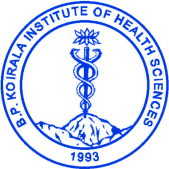


Participant Identification number for the study: Date seen:

Title of the study: Family Caregiver experience of caring COVID-19 patients admitted in COVID-19 hospital of a tertiary care hospital in Nepal.

I am Ms. Prekshya Thapa, Senior instructor (Faculty), Department of Psychiatric Nursing in the College of Nursing (CON), B.P. Koirala Institute of Health Sciences. The aim of this study is to understand experiences of caregivers caring for COVID-19 relatives during the hospitalization period. Participation in the study will enable us to obtain detailed information regarding human interactions and lived experiences of caregivers of COVID-19 patients and can be used to explore the perceived meaning and challenges of these experiences. This could help in further understanding of the needs of family caregivers and benefit policy makers to offer family caregivers greater support during and beyond the pandemic.

Your participation in this research is completely voluntary and so you are free to decide whether to take part or not. If you decide to take part, you will be given this information sheet to keep and be asked to sign a consent form for participating in the research. If you decide to take part, you are still free to withdraw at any time without giving a reason. You may decide not to take part at all. If you agree to participate, I will ask you to please sign this form before we can begin. If you are not able to sign, I will ask you for a thumbprint.

| Participant’s name:  ________________________ | Signature:  ___________________ | Date:  __________ |
| --- | --- | --- |
| Researcher’s name:  ________________________ | Signature:  ___________________ | Date:  __________ |

**Thumb print of participant**


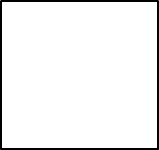


सहभागी सुसूचित मन्जुरी फारम

**अध्ययनका निम्ति सहभागी परिचय संख्या**: **मिति:**

**अध्ययनको शीर्षक: बी.पी. कोइराला स्वास्थ्य विज्ञान प्रतिष्ठान धरानको कोभिड अस्पतालमा भर्ना भएका बिरामीहरूको स्याहार गर्दाको परिवारको अनुभव :गुणात्मक अध्ययन**

म प्रेक्षा थापा र हाल वीपी कोइराला स्वास्थ्य विज्ञान प्रतिष्ठानको नर्सिङ कलेजमा senior instructor to रुपमा कार्यरत छु | यो अध्ययनको उदेश्य आफ्ना कोभिड-१९ संक्रमित आफन्त अस्पतालमा भर्ना हुँदाको बखत हेरचाह गर्दा भएको पारिवारिक स्याहारकर्ताहरुको मनोवैज्ञानिक अनुभवको अन्वेषण गर्नु हो | यस अध्ययनमा तपाईंको संलग्नताले हामीलाई कोभिड-१९ का बिरामीको पारीवारिक स्याहारकर्ताहरुको मानवीय अन्तरक्रिया तथा अनुभवको बारेमा विस्तृत जानकारी मिल्नेछ जसबाट हामीले यी अनुभवहरूको अर्थ एवं चुनौतीहरूको बारेमा अन्वेषण गर्न सक्नेछौं | यसले हामीलाई परिवारका हेरचाहकर्ताहरुको आवश्यकताको बारेमा बुझ्न मदत पुग्ने तपाई पुग्नेछ भने नीति निर्माताहरुलाई परिवारका हेरचाहकर्ताहरुलाई यस्तो महामारीमा र त्यसपश्चात पनि थप मदत उपलब्ध गराउन सहयोग गर्नेछ|

तपाईंले स्वेच्छाले सहभागी हुन मन्जूर गर्नु भए पछि केही प्रश्नहरु सोधिनेछ र त्यसको निम्ति ४५-६० मिनेट समय लाग्न सक्छ | यदि तपाईंले सहभागी हुन मन्जूर गर्नुभएमा शुरु गर्नु अघि म तपाईंलाई यो फारम सही गर्न अनुरोध गर्छु | यदि तपाईंले सही गर्न सक्नुहुन्न भने म औंठा छापको निम्ति अनुरोध गर्छु | यदि तपाईंले मन्जूरी दिन सक्नुहुन्न भने हामी तपाईंको नजिकको नातेदारलाई सहमतिको निम्ति अनुरोध गर्नेछौं |

| सहभागीको नाम:  ________________________ | हस्ताक्षर:  ___________________ | मिति:  __________ |
| --- | --- | --- |
| अनुसन्धानकर्ताको नाम:  ________________________ | हस्ताक्षर:  ___________________ | मिति:  __________ |
| हेरचाहकर्ताको नाम:  ________________________ | हस्ताक्षर:  ___________________ | मिति:  __________ |

सहभागीको औंठा छाप**:**


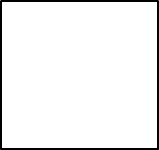

Supplement: S1 Appendix — (DOCX) [file pone.0295395.s002.docx]
