# Supplementary material for: Family caregiver experience of caring COVID-19 patients admitted in COVID-19 hospital of a tertiary care hospital in Nepal
Source: PLoS One. 2024 Jan 5;19(1):e0295395. doi: 10.1371/journal.pone.0295395 (PMC10769026; doi:10.1371/journal.pone.0295395)
Supplement: S2 Appendix — (DOCX) [file pone.0295395.s003.docx]

# S5 Appendix. According to COREQ 32-item checklist

Tong A, Sainsbury P, Craig, J. Consolidated criteria for reporting qualitative research (COREQ): a 32-item checklist for interviews and focus groups. Int J Qual Health Care. 2001;19(6): 349-357. doi:10.1093/intqhc/mzm042.

| **No.** | **Item** | **Guide**  **questions/descriptions** | **Reported on page** |
| --- | --- | --- | --- |
|  | Domain1:  Research team and reflexivity | | |
|  | **Personal Characteristics** | | |
| 1. | Interviewers/facilitators | Which author/s conducted the interview or focus group? | The first author (PT) conducted the interviews together with a data collection team.  Page no.7-8 |
| 2. | Credentials | What were the researcher’s credentials? | Page no. 5-6 |
| 3. | Occupation | What was their occupation at the time of the study? | Page no.5-6 |
| 4. | Gender | Was the researcher male or female? | (PT) female, (SL) female (NP) female, (GS)female, (MB) male, (NS) male,  (PU) male |
| 5. | Experience and training | What experience or training did the researcher have? | (PT) has experience in conducting qualitative study  All the coauthors have substantial experience in conducting research.  Page no.5-6 |
|  | **Relationship with participants** | | |
| 6. | Relationship established | Was a relationship established prior to study commencement? | No, the researchers did not know or meet any of the participants. Pageno.6 |
| 7. | Participant knowledge of the interviewer | What did the participants know about the researcher/s? | Participant information sheet was distributed to the participants about the study’s aim, design, and research questions as well about the researcher. |
| 8. | Interviewer characteristics | What characteristics were reported about the interviewer/facilitator? | Participants were informed about the researchers’ credentials, occupations, and clinical experience and contact number was shared. |
|  | Domain 2:  study design | | |
|  | **Theoretical framework** | | |
| 9. | Methodological orientation and Theory | What methodological orientation was stated to underpin the study? | .Page no. 6 |
|  | **Participant selection** | | |
| 10. | Sampling | How were participants selected? | Page no.7 |
| 11. | Method of approach | How were participants approached? | Page no. 7-8 |
| 12. | Sample size | How many participants were in the study? | Page no. 6-7 |
| 13. | Non‐participation | How many people refused to participate or dropped out? Reasons? | All caregivers requested agreed to participate in the study. |
|  | **Setting** | | |
| 14. | Setting of data collection | Where was the data collected? | Page no.7 |
| 15. | Presence of non-participants | Was anyone else present besides the participants and researchers? | Yes, other caregivers were present in the room and the data collection team. |
| 16. | Description of sample | What are the important characteristics of the sample? | The study participants were caregivers who had experience of attending their relatives in Covid caregiver residential facility. Page no.7 |
|  | **Data collection** | | |
| 17. | Interview guide | Were questions, prompts, guides provided by the authors? Was it pilot-tested? | Yes, we used a semi-structured interview guide  The interview guide was pilot-tested. Page no. 7-8 |
| 18. | Repeat interviews | Were repeat interviews carried out? | None. |
| 19. | Audio/visual recording | Did the research use audio or visual recordings to collect the data? | The interviews were conducted face to face and were audio recorded. Page no. 7-8 |
| 20. | Fieldnotes | Were field notes made during and/or after the interview? | Yes, field notes were taken by (RT) and (PU) and were used to ensure the details of the transcripts, including non-verbal communication.  Page no. 7-8 |
| 21. | Duration | What was the duration of the interviews | Page no.7-8 |
| 22. | Data saturation | Was data saturation discussed? | Page no. 7 |
| 23. | Transcripts returned | Were transcripts returned to participants for comment and/or correction? | Yes  Page no.9 |
|  | Domain 3:  analysis and findings | | |
|  | **Data analysis** | | |
| 24. | Number of data coders | How many data coders coded the data? | MB and PT |
| 25. | Description of the coding tree | Did the authors provide a description of the coding tree? | Yes, Fig1. |
| 26. | Derivation of themes | Were themes identified in advance or derived from the data? | They were derived from the data – inductive. |
| 27. | Software | What software, if applicable, was used to manage the data? | World and excel were used in sorting the data |
| 28. | Participant checking | Did participants provide feedback on the findings? | Yes. Page no.9-10 |
|  | **Reporting** | | |
| 29. | Quotations presented | Were participant quotations presented to illustrate the themes/findings? Was each quotation identified? | Yes |
| 30. | Data and findings consistent | Was there consistency between the data presented and the findings? | Yes |
| 31. | Clarity of major themes | Were major themes clearly presented in the findings? | Yes |
| 32. | Clarity of minor themes | Is there a description of diverse cases or discussion of minor themes? | Yes |
