# Supplementary material for: Family caregiver experience of caring COVID-19 patients admitted in COVID-19 hospital of a tertiary care hospital in Nepal
Source: PLoS One. 2024 Jan 5;19(1):e0295395. doi: 10.1371/journal.pone.0295395 (PMC10769026; doi:10.1371/journal.pone.0295395)
Supplement: Attachment — Submitted filename: Response to Reviewers.docx [file pone.0295395.s004.docx]

To,

The Academic Editor

Kshitij Karki

PLOS ONE Journal

Dear Dr. Karki,

Thank you for your reply regarding our manuscript “PONE-D-23-21744” entitled “Family Caregiver experience of caring COVID-19 patients admitted in COVID-19 hospital of a tertiary care hospital in Nepal”.

We are grateful for yours and reviewer’s insightful comments, and positive evaluation of our work. We have revised and modified text and the manuscript according to the critiques. As a consequence, we have added limitations, implications and added many new and clarifying statements in all parts of paper. The details of the response to editor comments along with the changes in manuscript is attached in the table at bottom of this letter. We have attached the manuscript with the track changes and the final manuscript as requested.

We hope these changes have improved the manuscript considerably and we hope it can be published without delay.

We look forward to your positive feedback and publication of manuscript in the reputed PLOS ONE Journal.

Yours Sincerely,

Prekshya Thapa

Senior Instructor, Department of Psychiatric Nursing

B.P. Koirala Institute of Health sciences, Dharan, Nepal

Email: [prekshya.thapa90@gmail.com](mailto:prekshya.thapa90@gmail.com)

The following changes has been made on the manuscript as per the comment.

| S.N. | Comments | Response to comment | Revised Manuscript (Pg.no, Section) |
| --- | --- | --- | --- |
| 1. | Please ensure that your manuscript meets PLOS ONE's style requirements, including those for file naming. | Thank you for the feedback. We have made necessary corrections as per the PLOS style requirements. | All text |
| 2. | Please include a copy of Tables 1 and 2 which you refer to in your text on pages 8 and 10. | Thank you for the feedback. The copy of Table 1 and 2 has been added to the text. | Section: Data Collection Page No.8 |
| 3. | We notice that your supplementary figures are uploaded with the file type 'Figure'. Please amend the file type to 'Supporting Information'. Please ensure that each Supporting Information file has a legend listed in the manuscript after the references list. | Thank you for the feedback. We have amended the file type figure to supporting information. | Page no. |
| 4. | Thank you for conducting this study. Many literature are there in Nepal and internationally regarding this issue. Please go through your data and try to generate the new themes and also consider the post covid situation as the reviewer questioned. I would like to see the limitations as well for the study and what the study will add the value in the evidence / or policy. | Thank you for this constructive feedback. We have added a new themes and limitations as well as implication of the study. | Themes has been revised in all domains.  Section: Strengths and Limitations Page No.27 and 28 |
| 5. | Abbreviations have been used in methodology without mentioning full word such as PT, RT, PU, GR, SL and NP, MB, NS. | The full name has been mentioned at first before using as abbreviated form. | Page no.5  Section Research team and reflexivity |
| 6. | Language editing of text is required. | The editing of the manuscript has been done as necessary. | All text of manuscript. |
| 7. | Although this study provides useful information about the experiences of informal caregivers of Covid-19 patients, it does not offer unique experiences that add to the qualitative findings of previous studies in this regard. The methodology and findings of this study are similar to the previous studies conducted in this field, and the authors did not mention much innovation in the study. The themes and sub-themes obtained in this study are almost identical to those found in previous studies. Given the time that has passed since the Covid-19 pandemic and the time of conducting this study, it was expected that more different data would be obtained. | Thank you so much for the feedback. We have made some changes in themes and sub-themes and had added the unique contribution of the study to policy and practice. | Page no. 28  Section: Implications to Policy and Practice. |
